# 'Virtual Mentorship is a No-Brainer': The Application of a Virtual Mentorship Programme for Prospective Plastic Surgery Trainees

**DOI:** 10.7759/cureus.73047

**Published:** 2024-11-05

**Authors:** Lucinda Z Motie

**Affiliations:** 1 Plastic Surgery, St Thomas' Hospital, London, GBR

**Keywords:** junior doctor, medical student, mentorship in surgery, plastic and reconstructive surgery, plastic surgery education, virtual mentorship

## Abstract

Aims

This study aimed to evaluate the effectiveness of a virtual mentorship programme in plastic surgery designed for medical students and foundation doctors in the United Kingdom. The programme sought to enhance understanding of common and emergency conditions, provide guidance on the application process for speciality training, and facilitate networking opportunities.

Materials and methods

The programme consisted of six sessions delivered via Microsoft Teams (Microsoft® Corporation, Redmond, WA) over a four-month period from May to August 2024. Participants completed online pre- and post-mentoring questionnaires. Wilcoxon signed-rank test was used to compare paired data responses.

Results

Ten participants completed both questionnaires; 90% were medical students, and 10% were foundation-year doctors. There was a significant increase in the understanding of common plastic surgery conditions and emergencies (p < 0.05), as well as improved knowledge of the application processes for core surgical training (p < 0.05) and higher speciality training (p < 0.05). Interest in the speciality significantly increased (p < 0.05), and participants were more likely to seek in-person mentorship (p < 0.05). The programme was well-received, with 100% rating it as 'excellent' or 'very good’.

Conclusions

The virtual mentorship programme effectively enhanced foundational knowledge, career preparation, and mentor-mentee relationships. Its implementation is recommended both alone and in combination with traditional face-to-face mentorship.

## Introduction

Plastic surgery is one of the most poorly taught specialities within the undergraduate medical curriculum in the United Kingdom (UK) [1]. With little to no exposure to medical school, those who are interested may find themselves without adequate support networks and mentorship within the field.

Mentorship has long been recognised as a crucial factor in speciality selection and career success in surgery [2]. This is especially true for women, who are less likely to pursue surgical careers due to the scarcity of female colleagues and role models [3]. Similarly, underrepresented minorities are more inclined to consider a surgical career when they encounter role models from similar ethnic backgrounds [4]. The absence of mentorship, coupled with negative societal stereotypes of plastic surgery often portrayed by the media [5], can discourage talented individuals from entering the field, resulting in a loss of promising candidates.

The COVID-19 pandemic saw rapid digital advancements in medical education. In the post-pandemic era, video-conference platforms such as Zoom (Zoom Video Communications, Inc., San Jose, CA) and Microsoft Teams (Microsoft Corporation®, Redmond, WA) remain a fundamental part of delivering modern education [6]. Students appreciate the ease of use and accessibility, irrespective of geographical location, increased student engagement and the ability to record among the many advantages of virtual education and digital platform use [6,7].

The global pandemic also accelerated the digitalisation of mentorship in plastic surgery. Particularly in America, where sub-internships and letters of recommendation play a key role in securing residency programmes, many medical schools incorporated mentorship into their virtual curricula [8,9]. These initiatives were well received, with one study reporting that nearly 20% of medical students would choose a virtual sub-internship over an in-person one [9]. Furthermore, medical students are increasingly seeking innovative platforms such as social media, especially Twitter, as alternative avenues for mentorship [10-13].

Four years on from the COVID-19 pandemic, the UK remains behind in well-established mentorship in plastic surgery despite widespread advancements in technology. This article presents a qualitative, survey-based study in which predominantly UK medical students interested in plastic surgery participated in a virtual mentorship programme, with the aim to provide guidance and greater insight into this underexposed surgical speciality.

## Materials and methods

The virtual mentorship programme was designed for medical students and foundation doctors in the UK. Its objectives were to (1) provide didactic teaching on common and emergency conditions in plastic surgery, (2) offer guidance on the application process for Core Surgical Training (CST) and higher speciality training (ST3), and (3) facilitate networking opportunities with junior and middle-grade plastic surgeons. To achieve these aims, the author developed six sessions. The programme was part of an online mentorship-enrichment scheme organised by a national medical society. It was advertised through the society’s social media platforms and weekly newsletter, and participants registered via an online form. The programme was carried out using the video-conference platform, Microsoft Teams, over a four-month period from May to August 2024. The programme included a mixture of didactic and case-based teaching sessions, networking opportunities in the form of question and answer, and informal drop-in sessions with a junior surgical trainee (senior house officer) and a higher speciality trainee (registrar). 

Participants completed a voluntary, self-reported questionnaire using Google Forms (Google, Inc., Mountain View, CA) before the first session, pre-mentoring, and after the final session, post-mentoring (see Appendix). Responses were collected using a five-point Likert scale (1 = poor; 5 = excellent or 1 = very unlikely; 5 = very likely) or a trichotomous scale (yes, no, maybe). Where relevant, free-response boxes were included to allow the participant to provide additional comments. Microsoft Excel 2013 (Microsoft Corporation®, Redmond, WA) with XLSTAT extension (Addinsoft, Paris, France) was used for data analysis. Wilcoxon signed-rank test was used to compare non-parametric paired data from pre- and post-mentoring responses, with significance set at p < 0.05.

## Results

Participant demographics

Ten participants completed both the pre- and post-programme questionnaires, with 90% being medical students and 10% being foundation-year doctors (Table 1).

**Table 1:** Participant demographics

Stage of training	n (%)
Pre-clinical medical student	1 (10)
Clinical year medical student	8 (80)
Foundation year doctor	1 (10)
Other	0 (0)

Plastic surgery was not part of their medical school curricula, and 80% of them had no prior mentorship in plastic surgery (Table 2).

**Table 2:** Additional pre-programme questionnaire results, data given as n (%)

	Yes (%)	No (%)	Maybe (%)
Are you interested in plastic surgery?	10 (100)	0 (0)	0 (0)
Is plastic surgery included in your medical school curriculum?	0 (0)	9 (90)	1 (10)
Have you had any prior mentorship in plastic surgery?	2 (20)	8 (80)	0 (0)
Do you think mentorship would affect whether you pursue a career in plastic surgery?	10 (0)	0 (0)	0 (0)

Knowledge of clinical conditions and training pathways 

Post-programme, there was a significant increase in understanding of common conditions and emergencies (p < 0.05), as well as the application process for CST (p < 0.05) and ST3 (p < 0.05) (Table 3).

**Table 3:** Pre- and post-programme questionnaire results, data given as n (%)

	Pre- or post-programme	1 - Poor (%)	2 - Fair (%)	3 - Good (%)	4 - Very good (%)	5 - Excellent (%)	p-value
How well do you understand common conditions and emergencies in plastic surgery?	Pre	0 (0)	5 (50)	4 (40)	1 (10)	0 (0)	0.007
	Post	0 (0)	0 (0)	1 (10)	5 (50)	4 (40)	
How well do you understand the application process for Core Surgical Training?	Pre	1 (10)	2 (20)	0 (0)	5 (50)	2 (20)	0.023
	Post	0 (0)	0 (0)	1 (10)	3 (30)	6 (60)	
How well do you understand the application process for ST3 Plastic Surgery?	Pre	3 (30)	5 (50)	2 (20)	0 (0)	0 (0)	0.004
	Post	0 (0)	0 (0)	1 (10)	6 (60)	3 (30)	

Interest in plastic surgery 

Interest in the speciality increased significantly from 50% to 100% of participants indicating they were likely or very likely to pursue a career in plastic surgery (p < 0.05) (Table 4).

**Table 4:** Pre- and post-programme questionnaire results continued, data given as n (%)

	Pre- or post-programme	1 - Very unlikely (%)	2 - Unlikely (%)	3 - Neither (%)	4 - Likely (%)	5 - Very likely (%)	p-value
How likely are you to pursue a career in plastic surgery in the future?	Pre	0 (0)	1 (10)	4 (40)	3 (30)	2 (20)	0.014
	Post	0 (0)	0 (0)	0 (0)	4 (40)	6 (60)	
How likely are you to seek out in-person mentorship in plastic surgery?	Pre	1 (10)	0 (0)	4 (40)	3 (30)	2 (20)	0.016
	Post	0 (0)	0 (0)	0 (0)	4 (40)	6 (60)	

Mentorship interest 

Post-programme, all participants reported they were likely or very likely to seek in-person mentorship, a significant rise from 50% pre-programme (p < 0.05) (Table 4). All participants agreed that mentorship would influence their career choice of plastic surgery (Table 2) and expressed support for more virtual mentorship opportunities in plastic surgery (Table 5).

**Table 5:** Additional post-programme questionnaire results, data given as n (%)

	Yes (%)	No (%)	Maybe (%)
Should there be more virtual mentorship programmes in plastic surgery?	10 (100)	0 (0)	0 (0)

Overall programme satisfaction

All participants rated the programme as 'excellent' or 'very good' (Table 6).

**Table 6:** Post-programme rating of virtual mentorship programme, data given as n(%)

	Poor (%)	Fair (%)	Good (%)	Very good (%)	Excellent (%)
How would you rate this virtual mentorship programme?	0 (0)	(0)	(0)	4 (40)	6 (60)

## Discussion

The programme was well received with all participants expressing support for more virtual mentorship opportunities in plastic surgery. Interactive features such as polls, quizzes, and breakout rooms were incorporated into the programme and have been shown to enhance engagement and learning outcomes [14]. The virtual format also enabled students from across the country to participate at times convenient to them, eliminating travel costs and reflecting the broader benefits of online learning, as seen in the literature [7]. One participant also commended the use of teleconference platforms for mentorship, stating, *‘Mentorship should be a cornerstone of any medical speciality training. With facilities like Zoom and Teams, virtual mentorship is a no-brainer’*.

Virtual mentorship helps minimise the exposure gap created by varying undergraduate curricula across the UK. Similar to reported literature [1], participants had limited prior exposure to plastic surgery before joining the programme. One participant noted how this lack of exposure influenced their career decision-making, *‘If I were to get a better idea of what plastic surgery is like, then I would know whether or not it seems like a viable career option for me. As of right now, I've no experience, so couldn't say whether I would like it or not yet’*. After the programme, there was a significant increase in both interest and theoretical knowledge of the speciality. Virtual mentorship can, therefore, provide a foundational understanding of the speciality whilst helping to retain talented potential trainees who might have otherwise been deterred by insufficient exposure. 

Exposure to plastic surgery is useful even for those pursuing other specialities. Recognising and managing common plastic surgery conditions such as burns, devascularised limbs, amputations, and flexor sheath infections is relevant to several non-surgical specialities, including general practice and emergency medicine [1]. Diminished exposure may exacerbate practice differences in the future with the potential to adversely affect patient care. 

Plastic surgery is one of the most competitive specialities for higher surgical training [15], making it imperative for aspiring trainees to have a clear understanding of the application process. Concerns have been raised by Pasha et al. [16] about the disconnection between perceptions of the competitive national selection process and awareness of what the application involves. The mentorship programme significantly increased understanding of the application process for both junior and higher surgical training. Virtual mentorship provides a solution to this concern, helping prospective trainees better prepare for their careers. 

The mentorship programme also facilitated interactions between participants and surgeons at different stages of training, including both junior and higher surgical trainees. This multi-level mentorship structure offered participants deeper insights into the speciality and allowed them to build networks with established professionals in the field. Importantly, this led to the formation of a ‘mentorship team’, reducing the risk of ‘mentorship malpractice’, a concept introduced by Chopra et al. [17] to describe the over-reliance on a single mentor and the potential for dysfunctional mentor-mentee relationships. The ease of collaboration through online platforms further reduces the risks of suboptimal mentoring. Additionally, combining virtual mentorship with traditional in-person mentoring could offer a well-rounded approach to combating mentorship malpractice.

Despite the positive feedback, one participant expressed interest in expanding the programme to include in-person mentorship, stating, ‘Considering the virtual and long-distance nature of the scheme, there isn't much else that can be done... try and expand into an in-person mentorship scheme too!’ This sentiment aligns with findings from Kidess et al. [18], wherein students desired a face-to-face event with practical exercises despite a successful virtual internship day. Whilst virtual mentorship enables a range of opportunities, it is unlikely to fully replace traditional face-to-face mentorship, particularly in a surgical speciality where hands-on skills are essential and difficult to simulate online. Notably, procedural aspects of plastic surgery were not covered in the virtual programme. However, the programme did significantly increase the likelihood of participants seeking in-person mentorship post-programme. Virtual mentorship can, therefore, serve as an introductory experience that fosters interest and encourages participants to explore the field further through in-person experiences. 

Limitations

Whilst findings were significant, the author acknowledges several limitations. Data were collected from a relatively small cohort of participants, making it difficult to generalise the results to a larger population. The participants were a self-selected group with a specific interest in plastic surgery, introducing an inherent selection bias. It is unclear how the virtual mentorship sessions would impact medical students and junior doctors who are undecided or have no prior interest in the field. The self-selection of motivated participants, who actively sought mentorship outside of compulsory education or work, may have excluded individuals with differing levels of interest or awareness of the programme, thereby limiting the diversity of perspectives. An extended timeframe may be necessary to gather longitudinal evidence on career uptake in plastic surgery. Furthermore, the outcomes of virtual mentorship were not compared with traditional in-person mentorship or a control group, which would be necessary to fully evaluate its effectiveness. Future studies should address these limitations to better assess the true impact of virtual mentorship programmes. 

## Conclusions

Mentorship is fundamental to career success for aspiring surgical trainees. However, undergraduate curricula in the UK do not favour the field of plastic surgery or its prospective trainees. This study demonstrates the effectiveness of a virtual mentorship programme in providing a strong foundational understanding of the speciality, enabling prospective trainees to better prepare for their careers and fostering meaningful mentor-mentee relationships. Although virtual mentorship is unlikely to fully replace traditional face-to-face mentorship, it offers an innovative and complementary approach that can aid career decision-making and advancement in plastic surgery whilst motivating prospective trainees to seek hands-on experiences. Going forward, we strongly encourage universities and national professional bodies to embrace virtual mentorship programmes and to adopt a hybrid approach to mentorship, similar to the blended learning strategies prevalent in medical education today.

## Appendices

Pre-programme questionnaire  

What year are you in? 

Pre-clinical 

Clinical year 1 

Clinical year 2 

Final year 

Other 

Are you interested in plastic surgery? 

Yes 

No 

Maybe 

Is plastic surgery included in your medical school curriculum? 

Yes 

No 

Maybe 

Have you had any prior mentorship in plastic surgery? 

Yes 

No 

If you answered yes, please briefly describe what mentorship you have received.

Do you think mentorship would affect whether you pursue a career in plastic surgery? 

Yes 

No 

Maybe 

Please expand further on your answer to the above question if you wish.

How well do you understand common conditions and emergencies in plastic surgery? 

1. Poor 

2. Fair 

3. Good 

4. Very good 

5. Excellent  

How well do you understand the application process for Core Surgical Training? 

1. Poor 

2. Fair 

3. Good 

4. Very good 

5. Excellent  

How well do you understand the application process for ST3 Plastic Surgery? 

1. Poor 

2. Fair 

3. Good 

4. Very good 

5. Excellent  

How likely are you to pursue a career in plastic surgery in the future? 

1. Very unlikely 

2. Unlikely 

3. Neither 

4. Likely 

5. Very likely 

How likely are you to seek out in-person mentorship in plastic surgery? 

1. Very unlikely 

2. Unlikely 

3. Neither 

4. Likely 

5. Very likely 

Post-programme questionnaire  

How well do you understand common conditions and emergencies in plastic surgery? 

1. Poor 

2. Fair 

3. Good 

4. Very good 

5. Excellent  

How well do you understand the application process for Core Surgical Training? 

1. Poor 

2. Fair 

3. Good 

4. Very good 

5. Excellent  

How well do you understand the application process for ST3 Plastic Surgery? 

1. Poor 

2. Fair 

3. Good 

4. Very good 

5. Excellent  

How likely are you to pursue a career in plastic surgery in the future? 

1. Very unlikely 

2. Unlikely 

3. Neither 

4. Likely 

5. Very likely 

How likely are you to seek out in-person mentorship in plastic surgery? 

1. Very unlikely 

2. Unlikely 

3. Neither 

4. Likely 

5. Very likely 

How would you rate this mentorship programme? 

1. Poor 

2. Fair 

3. Good 

4. Very good 

5. Excellent 

How would you improve this mentorship series? 

Should there be more virtual mentorship programmes in plastic surgery? 

Yes 

No  

Maybe 

Please expand further on your answer to the above question if you wish.
